# Combined iodine-125 seed strand, portal vein stent, transarterial chemoembolization, lenvatinib and anti-PD-1 antibodies therapy for hepatocellular carcinoma and Vp4 portal vein tumor thrombus: A propensity-score analysis

**DOI:** 10.3389/fonc.2022.1086095

**Published:** 2023-01-19

**Authors:** Zi-Han Zhang, Si-Nan Hou, Jia-Ze Yu, Wen Zhang, Jing-Qin Ma, Min-Jie Yang, Qing-Xin Liu, Ling-Xiao Liu, Jian-Jun Luo, Xu-Dong Qu, Zhi-Ping Yan

**Affiliations:** ^1^ Department of Interveintional Radiology, Zhongshan hospital, Fudan, University, Shanghai, China; ^2^ Shanghai Institute of Medical Imaging, Fudan University, Shanghai, China; ^3^ National Clinical Research Center of Interventional Medicine, Zhongshan Hospital, Fudan University, Shanghai, China

**Keywords:** hepatocellular carcinoma, portal vein tumor thrombus, iodine-125 seed strand, transarterial chemoembolization, lenvatinib, anti-PD-1 antibody

## Abstract

**Objective:**

To evaluate the safety and efficacy of interventional therapy (iodine-125[^125^I] seed strand and portal vein stent [PVS] implantation plus transarterial chemoembolization [TACE]) combined with systemic therapy (lenvatinib plus anti-PD-1 antibody) as first-line treatment for hepatocellular carcinoma (HCC) patients with Vp4 portal vein tumor thrombus (PVTT).

**Patients and methods:**

From December 2018 to October 2021, 87 HCC patients with Vp4 PVTT were included in this single-center retrospective study. Forty-seven patients underwent interventional therapy combined with lenvatinib and anti-PD-1 antibody (group A), while 40 cases underwent interventional therapy combined with lenvatinib only (group B). Overall response rate (ORR), stent occlusion rates (SOR), median overall survival (OS), median progression-free survival (PFS) and median stent patency time (SPT) were compared between the 2 groups.

**Results:**

The mean intended dose (r = 10 mm; z = 0; 240 days) was 64.9 ± 1.0 Gy and 64.5 ± 1.1 Gy in group A and B, respectively (*p* = 0.133). ORR and SOR were significantly different between group A and B (ORR, 55.3% vs 17.5%, *p* < 0.001; SOR, 12.8% vs 35.0%, *p* = 0.014). In the propensity-score matching (PSM) cohort, the median OS, median PFS and median SPT were significantly longer in group A compared with group B (32 PSM pairs; OS, 17.7 ± 1.7 vs 12.0 ± 0.8 months, *p* = 0.010; PFS, 17.0 ± 4.3 vs 8.0 ± 0.7 months, *p* < 0.001; SPT, not-reached vs 12.5 ± 1.1 months, *p* = 0.028).

**Conclusion:**

This interventional therapy combined with lenvatinib and anti-PD-1 antibody is safe and effective for HCC patients with Vp4 PVTT.

## Introduction

Portal vein tumor thrombus (PVTT), a common pattern in advanced hepatocellular carcinoma (HCC), is found in 10~40% of patients (1). The prognosis of patients with PVTT in the main trunk (Vp4 PVTT) remains poor. The median overall survival (OS) of these patients is only 2.7~4.0 months if untreated (2). The perioperative mortality rate is 0%-28%, with a 5-year OS rate of 0%-26% (3, 4).

Based on the SHARP and REFLECT trials (5, 6), sorafenib and lenvatinib were recommended as first-line systemic therapy for patients with advanced unresectable HCC (7). However, Kaneko et al. reported a median OS of only 5.5 months in patients with Vp3/4 PVTT administered sorafenib and Lenvatinib (8).

Linear iodine-125(^125^I) seed strand combined with portal vein stent (PVS) implantation plus transarterial chemoembolization (TACE) was proposed by Luo et al. for patients with HCC and Vp4 PVTT (9). Zhang et al. conducted a retrospective study that combined sorafenib with this interventional treatment strategy. This combined therapy prolonged the OS to 12.3 months in these patients (10).

In recent years, immune checkpoint inhibitor (ICI) therapy, particularly applying antibodies targeting the programmed cell death-1 (PD-1)/programmed cell death ligand-1 (PD-L1) pathway, has been a significant component of numerous combination regimens in advanced HCC (11–13).

This study performed to evaluate the safety and efficacy of interventional therapy (^125^I seed strand and PVS implantation plus TACE) combined with systemic therapy (lenvatinib plus anti-PD-1 antibody) as first-line treatment for HCC patients with Vp4 PVTT.

## Materials and methods

### Patients

**Figure 1:**
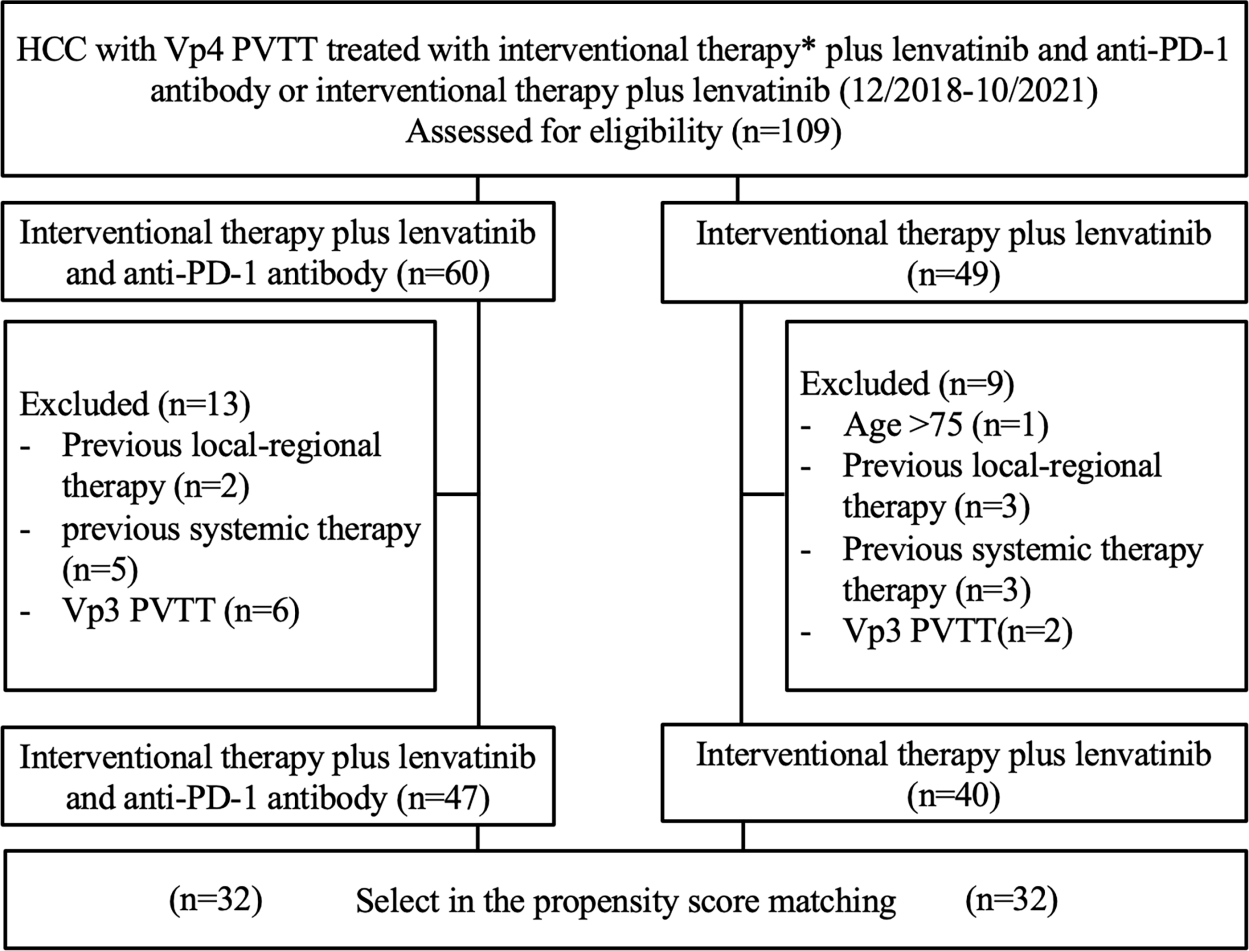
Patient selection flow chart. * Interventional therapy = ^125^I seed strand and PVS implantation plus TACE.

This was a single-center retrospective study. The study was approved by the local institutional review board. Informed consent was waived. We reviewed the electronic medical records of 109 consecutive patients with hepatitis B-related HCC and Vp4 PVTT, who were administered interventional therapy (^125^I seed strand and PVS implantation plus TACE) combined with systemic therapy (lenvatinib plus anti-PD-1 antibody) (group A) or interventional therapy (^125^I seed strand and PVS implantation plus TACE) combined with lenvatinib only (group B) from December 2018 to October 2021. Before treatment initiation, the benefits, and potential adverse events (AEs) related to both combination regimens were explained thoroughly to the patients. The final choices were made by the patients. (**Figure 1**)

Intrahepatic HCC was diagnosed based on the American Association for the Study of Liver Disease guidelines or histology (14). According to the standard recommended by Shah et al. (15), a PVTT was considered to be neoplastic if at least one of the following criteria was met: (a) expansion of the involved vessel (vessel diameter ≥ 1.8 cm for the MPV, ≥ 1.6 cm for the right portal vein (PV), or ≥ 1.8 cm for the left PV; (b) clear evidence of enhancement on dynamic contrast-enhanced CT images during the arterial phase of dynamic imaging, compared with baseline images (≥ 20 HU on CT). Otherwise, the PVTT was bland. The extent of PVTT was classified as follows: Vp0, no PVTT; Vp1, segmental PV invasion; Vp2, right anterior or posterior PV; Vp3, right or left PV; Vp4, main trunk and/or contralateral portal vein branch to the primarily involved lobe (16).

Inclusion criteria were: [1] between 18 and 75 years of age; [2] a single tumor ≥ 5.0 cm or multiple nodular tumors > 3.0 cm; [3] Vp4 PVTT; [4] patent second-order branch of the portal vein prior to PVTT; [5] Child-Pugh class A or B; and [6] an Eastern Cooperative Group performance status (ECOG) score of 0-2. These points represent eligibility criteria for the treatment.

Exclusion criteria were: [1] Vp1-3 PVTT; [2] completely occluded portal vein; [3] hepatic encephalopathy, severe ascites, esophageal, gastric fundal variceal bleeding or other serious medical comorbidities; [4]previous local-regional therapy (radiofrequency ablation [RFA], microwave ablation [MWA], cryoablation, yttrium-90 [90Y] radioembolization, stereotactic body radiotherapy [SBRT], hepatic artery infusion chemotherapy [HAIC], or liver transplantation); [5] previous systemic therapy (tyrosine kinase inhibitors [TKIs], systemic chemotherapy, or immunotherapy); or [6] malignant tumor other than HCC.

### Interventional therapy

The protocol for interventional therapy (^125^I seed strand and PVS implantation plus TACE) was the same in both groups.

### ^125^I seed properties

**Figure 2:**
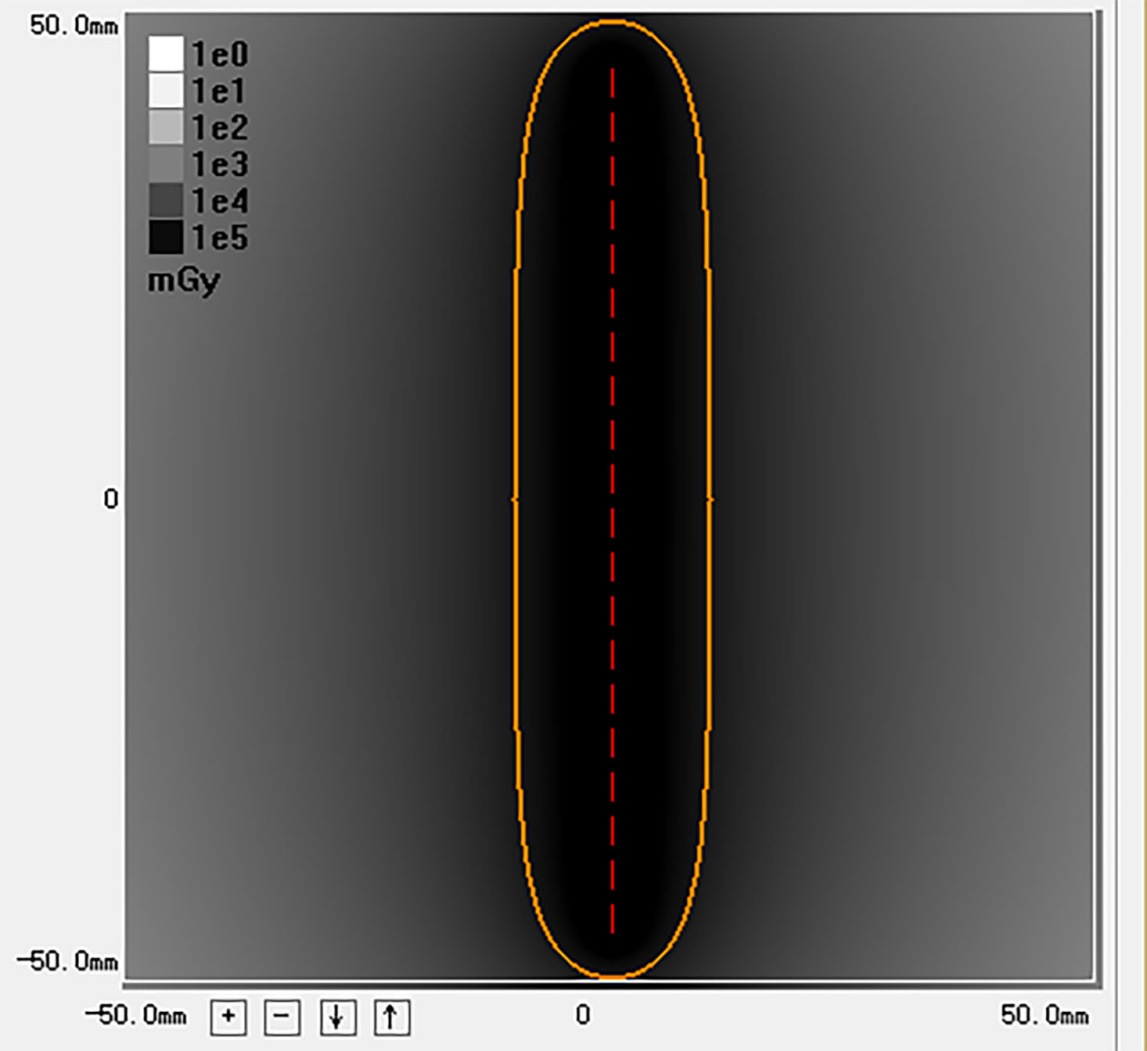
This image is the radiation distribution of a strand loaded with 20 ^125^I seeds simulated by the calculation software. The yellow circle represents a 100% isodose contour (r = 10mm). The 240 days’ intended dose of this ^125^I seed strand is 63.5 Gy.

Model 6711 ^125^I seeds (XinKe; Shanghai, China) were used in this study. The radioactivity of each ^125^I seed was 25.9 MBq with a half-life of 59.4 days. Principal photon emissions were 27.4 and 35.5 keV X-rays and gamma-rays, respectively. The half-value thickness of the tissue for ^125^I seed was 17 mm, and the incipient dose rate was 7 cGy/h. The 240-day intended dose at 10 mm from the axis of the ^125^I seed strand was calculated with a radiation calculation software (version 0.1) based on the American Association of Physicists in Medicine TG43U1 brachytherapy formalism (17). (**Figure 2**)

The production process of ^125^I seed strands was as follows: (a) a 4-F flexible compliant cannula (Boston Scientific, Natick, Massachusetts) was sealed at one end with an alcohol lamp; (b) ^125^I seeds were loaded into the tube linearly, and the number of ^125^I seeds loaded (N) was determined as N = L/4.5 + 4, where L (mm) is the length of the obstructed PV (9); (c) the other end was cut and sealed.

### ^125^I seed strand and PVS implantation

The contralateral second­order branch was punctured with a 21-gauge Chiba needle (Cook, Bloomington, Indiana) under ultrasound guidance, followed by the insertion of a 0.018-inch wire (Cook) into the MPV. A 6-F NEEF set (Cook) was introduced into the MPV over the wire. Through the outer cannula of the 6-F NEEF set, a 0.035-inch wire (Terumo, Tokyo, Japan) combined with a 4-F Cobra catheter (Cordis, Miami Lakes, Florida) was manipulated across the obstructed MPV into the superior mesenteric vein (SMV). The 4-F Cobra catheter and the 6-F NEEF set were removed, and a 6-F sheath (Cordis) was introduced through the wire. Portography was performed to measure the diameter and length of the obstructed MPV by a 4-F pigtail catheter (Cook) placed in the SMV. Two 0.035-inch stiff wires (Terumo) were inserted into the SMV through the 6­F sheath. After the sheath removal, the 6-F NEFF set and a self-expandable stent (Bard, New Jersey, America) of appropriate size were introduced into the MPV over one of the stiff wires, respectively. The stent was deployed from the distal MPV into the contralateral fist-order branch of the portal vein. A ^125^I seed strand was delivered to the target position *via* the outer cannula of the 6-F NEFF set and released between the stent and the MPV. Portography was repeated through the 4-F pigtail catheter (Cook). The puncture tract was next occluded by 3 × 140 mm Nester coils (Cook).

**Figure 3:**
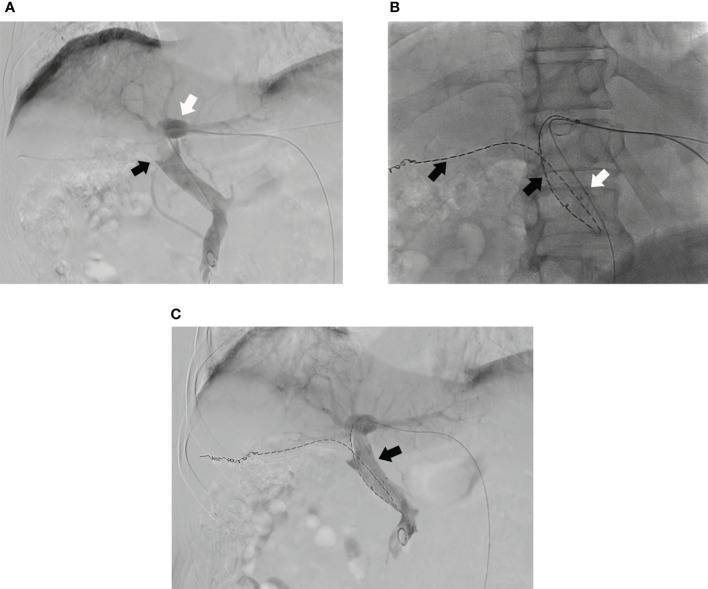
**(A)** Portography of a male patient in group A shows tumor thrombus in MPV (black arrow) and the left portal vein branch is still patent (white arrow) and; **(B)** A ^125^I seed strand (black arrow) and a stent (white arrow) are placed from left portal vein to MPV and another ^125^I seed strand is placed into right portal vein (black arrow); **(C)** The portal venography shows the MPV is more patent after the PVS and ^125^I seed strands implantation (black arrow).

Then, the ipsilateral second-order portal vein branch was punctured with a 21-gauge Chiba needle (Cook) under ultrasound guidance. With confirmed access, a 0.018-inch wire (Cook) was manipulated to cross the obstructed segment of ipsilateral portal vein branch and positioned into the MPV. A 6-F NEFF set (Cook) was introduced into the ipsilateral portal vein over the 0.018-inch wire. Then, the 0.018-inch wire was replaced by a 0.035-inch wire (Cook). Another ^125^I seed strand was pushed to the target position of PVTT in ipsilateral portal vein branch by the inner core of the 6-F NEFF set. Then, the outer cannula of the 6-F sheath was retreated slowly until the strand was completely released. The position of the strand should completely cover the macroaxis of PVTT in ipsilateral portal vein branch. Finally, the transhepatic puncture track was occluded by 3 × 140 mm Nester coils (Cook) (**Figures 3A-C**).

### TACE procedure

TACE was provided after the ^125^I seed strand and PVS implantation immediately. Hepatic angiography was performed to evaluate tumor vascularity. A chemotherapeutic emulsion consisting of 10-50 mg epirubicin (Pharmorubicin; Pfizer, New York) and 4-10 ml lipiodol (Lipiodol; Guerbet, Roissy, France) was slowly injected at a rate of 0.5-1.0 mL/min under fluoroscopic guidance *via* a 2.4-F microcatheter (Merit Medical, USA) until saturation of the tumor-supplying arteries. The dose of iodized oil was calculated as 1.0-1.5 ml per cm in dimeter of tumor. If the tumor had a rich blood supply, more oil was needed and vice versa. The dose of epirubicin was calculated as 10–50 mg/m^2^ of body surface area. Then, 350-560-μm gelatin sponge particles (Jingling, Jiangsu, China) were used to embolize the residual feeding artery of tumor.

### Systemic therapy

In both groups, all patients received Lenvatinib (MSD, USA) 3 days after the first interventional procedure. Lenvatinib was orally administered at 8 mg/day in patients weighing <60 kg and at 12 mg/day in those ≥60 kg. In patients developing AEs (grade ≥2), dose reduction or temporary interruption was maintained until the symptoms resolved to grade 0-1. AEs were assessed by the National Cancer Institute (NCI) Common Terminology Criteria for Adverse Events (CTCAE v4.03).

In group A, patients received anti-PD-1 inhibitor injection in 3-7 days after the first interventional procedure. They were monitored regularly, including repeat safety evaluation 2-3 days prior to each anti-PD-1 antibody treatment cycle. Anti-PD-1 antibodies were intravenously administered as follows: nivolumab (Bristol-Myers Squibb, USA) 3 mg/kg or camrelizumab (Hengrui Medicine, China) 200 mg every 2 weeks (18), or pembrolizumab (MSD, USA) 200 mg, sintilimab (Innovent Biologics, China) 200 mg (19) or toripalimab (Junshi Bioscience, China) 240 mg (20), every 3 weeks. In patients developing AEs (grade 2), temporary interruption was maintained until the symptoms resolved to grade 0-1. In patients developing AEs (grade 3-4), anti-PD-1 inhibitor injection was ceased permanently.

### Post-procedure management

Single photon-emission computer tomography combined with CT (SPECT/CT) was performed on day 1 to evaluate the location and distribution of radiation by the ^125^I seed strand. Laboratory tests (including hepatic and renal functions, complete blood count, and coagulation parameters) were performed 3-7 days after the initial procedure.

In the first 2 days, 4,100 U of low- molecular-weight heparin (XinYi, Shanghai, China) was injected subcutaneously twice a day. Beginning 3 days after the procedure, warfarin (XinYi, Shanghai, China), starting with 2.5 mg every day, was administered to all patients, and continued for 6 months. The dose of warfarin was adjusted based on the coagulability test (international normalized ratio = 1.8–2.0).

### Follow-up and evaluation

**Figure 4:**
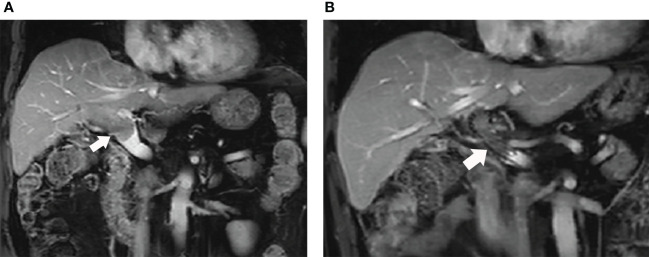
**(A)** The MRI images of this male patient shows the tumor thrombus had invaded into the MPV from right portal vein (white arrow); **(B)** The patient received ^125^I seed strands and PVS implantation plus TACE combined with lenvatinib and anti-PD-1 antibody therapy. The MRI images performed 11 months after the initial procedure shows the stent is still patent (white arrow).

The follow-up period was defined as the time from the initial interventional procedure to death or the last follow-up date. Each follow-up session included a detailed medical history, physical examination, laboratory tests, and contrast-enhanced CT or MRI. Follow-up was conducted every 30-45 days after the initial procedure. Patients with residual viable tumors or recurrent tumors in the hepatic parenchyma on CT or MRI images underwent repeated TACE in case the Child-Pugh status remained at class A or B. No other interventional therapy was provided except for TACE. (**Figures 4A, B**)

The primary endpoint was overall survival (OS, defined as the time from the initial interventional procedure to death from any cause). Secondary endpoint was progression-free survival (PFS, defined as the time from the initial interventional procedure until tumor progression or death from any cause).

Intrahepatic tumor response was classified as complete response (CR), partial response (PR), stable disease (SD), or progressive disease (PD) according to modified Response Evaluation Criteria in Solid Tumor (mRECIST) criteria. Overall response rate (ORR) was defined as the percentage of patients who had a best tumor response rating of CR or PR. Disease control rate (DCR) was defined as the percentage of patients achieving CR, PR or SD as the best tumor response.

PVTT response was evaluated by the rate of stent occlusion and the median stent patency time (SPT). Because PVTT was changed into an irregular shape and positioned between the stent and the portal vein wall after stent implantation, it is hard to calculate the volume of PVTT precisely. Stent occlusion was defined with no contrast medium detected inside the stent on the portal phase of contrast-enhanced CT or contrast-enhanced MRI images, or no blood flow signal detected by color doppler flow imaging (CDFI). SPT was determined from the day of stent placement to stent occlusion or the day of last follow-up.

### Statistical analysis

All statistical analysis was performed with SPSS (version 23.0, Chicago, Illinois). Continuous variables were presented as mean ± standard deviation and were compared by independent or paired samples *t* test. Categorical variables were presented as frequency and compared by the Chi-square test. Median PFS, median OS and median SPT were analyzed by the Kaplan-Meier method and the log-rank test. A *p*-value < 0.05 was considered statistically significant. Factors statistically significant at *p*-value < 0.05 in univariate analysis were entered a multivariable Cox proportional hazards model.

Sex, age, tumor size, Child-Pugh class, AFP level and extrahepatic metastasis were considered within the propensity-score matching (PSM) model. PSM was performed, with a matching ratio of 1:1 for both groups, using the nearest-neighbor matching method, with a caliper distance of 0.2 without replacement. OS, PFS, SPT and multivariate analysis were compared between the matched groups.

## Results

### Patients

According to the inclusion and exclusion criteria, 87 patients were included in this study (Group A, n=47; and Group B, n=40). Baseline characteristics are presented in (**Table 1**). After the PSM, 32 pairs were matched.

**Table 1:** Baseline characteristics of the 2 groups before and after propensity score matching.

	Before propensity score matching			After propensity score matching		
Characteristic	Group A (n=47)	Group B (n=40)	*p*-value	Group A (n=32)	Group B (n=32)	*p*-value
Sex			1.000			1.000
Male	42 (89.4)	36 (90.0)		29 (90.6)	30 (93.8)	
Female	5 (10.6)	4 (10.0)		3 (9.4)	2 (6.3)	
Age			0.425			0.448
≥55y	23 (48.9)	23 (57.5)		17 (53.1)	20 (62.5)	
<55y	24 (51.1)	17 (42.5)		15 (46.9)	12 (37.5)	
Tumor size *(mm)			0.446			1.000
≥10cm	22 (46.8)	22 (55.0)		16 (50.0)	16 (50.0)	
<10cm	25 (53.2)	18 (45.0)		16 (50.0)	16 (50.0)	
Extrahepatic metastasis			0.786			1.000
Yes	5 (10.6)	5 (12.5)		3 (9.4)	2 (6.3)	
No	42 (89.4)	35 (87.5)		29 (90.6)	30 (93.8)	
Child-Pugh class			1.000			1.000
A	44 (93.6)	38 (95.0)		30 (93.8)	30 (93.8)	
B	3 (6.4)	2 (5.0)		2 (6.3)	2 (6.3)	
ECOG performance status			0.658			0.238
0/1	45 (95.7)	37 (92.5)		32 (100.0)	29 (90.6)	
2	2 (4.3)	3 (7.5)		0 (0.0)	3 (9.4)	
Serum AFP level			0.064			0.784
≥400	25 (53.2)	29 (72.5)		23 (71.9)	22 (68.8)	
<400	22 (46.8)	11 (27.5)		9 (28.1)	10 (31.3)	

Values in parentheses are percentages.

AFP, α-fetoprotein; ECOG, Eastern Cooperative Oncology Group.

*Tumor size, the maximum diameter of the largest target index lesion.

### Technical success

The technique was performed successfully in all patients. The mean number of ^125^I seeds loaded were 38.0 ± 13.5 (range, 20-60) and 33.4 ± 14.7 (range, 16-60) in groups A and B, respectively (*p* = 0.136). The mean intended doses were 64.9 ± 1.0 Gy (range, 63.5-66.5 Gy) and 64.5 ± 1.1 Gy (range, 63.2-66.5 Gy) in groups A and B, respectively (*p* = 0.133). No dislodge of ^125^I seed strand was observed in SPETCT/CT and CT images. Totally 87 patients in both groups received a total of 296 TACE procedures (154 and 142 in groups A and B, respectively). Mean 3.3 ± 1.9 (range 1-9) and 3.6 ± 1.6 (range 1-8) TACE procedures were performed in groups A and B, respectively (*p* = 0.476).

### Tumor response

Treatment response for intrahepatic tumors in all patients is presented in (**Table 2**). ORR and DCR were significantly higher in group A compared with group B (ORR, 55.3% vs 17.5%, *p* < 0.001; DCR, 70.2 vs 30.0, *p* < 0.001).

**Table 2:** Response of intrahepatic HCC.

	Group A n=47	Group B n=40	*P*-value
CR	2	0	
PR	24	7	
SD	7	5	
PD	14	28	
ORR (%)	55.3	17.5	<0.001
DCR (%)	70.2	30.0	<0.000

CR, complete response; DCR, disease control rate; PD, progressive disease; PR, partial response; SD, stable disease.

ORR = (CR + PR)/n.

DCR = (CR + PR + SD)/n.

Stent occlusion by tumor invasion was observed in 6 (12.8%) group A and 14 (35.0%) group B patients (*p* = 0.014). The cumulative stent patency rates at 3-, 6-, 9- and 12-months were 97.8%, 93.3%, 88.7% and 88.7% in group A, and 100.0%, 89.5%, 81.6% and 76.3% in group B, respectively (*p* = 0.003).

In group A, 2 patients with PR tumor response were administered liver transplantation at 11 and 11.5 months after the initial interventional therapy, respectively. One patient in group A with PR tumor responses was administered surgical resection of intrahepatic tumor at 11.7 months after the initial interventional therapy. No patient received surgical resection or liver transplantation in group B.

### Survival

The mean follow-up times were 14.2 ± 5.1 and 11.0 ± 5.0 months in groups A and B, respectively. During the follow-up period, 25 (53.2%) and 34 (85.0%) patients died in groups A and B, respectively (*p* = 0.002). Overall survival rates at 3-, 6-, 9- and 12-months were 93.6%, 89.4%, 80.9% and 76.6% in group A, and 92.5%, 85.0%, 65.0% and 43.7% in group B, respectively (*p* < 0.001). The causes of death are presented in (**Table 3**).

**Table 3:** The causes of death in 2 groups.

Causes of death	Group A (n=25)	Group B (n=34)	*p*-value
Tumor progression	7(28.0)	19(55.9)	0.033
hepatic failure	7(28.0)	7(20.6)	0.508
Variceal bleeding	6(24.0)	5(14.7)	0.365
Hepatic encephalopathy	1(4.1)	1(2.9)	1.000
Liver abscess	0(0.0)	1(2.9)	1.000
Respiratory failure	1(4.1)	1(2.9)	1.000
Myocardial infarction	2(8.0)	0(0.0)	0.175
Cerebral hemorrhage	1(4.1)	0(0.0)	0.424

Values in parentheses are percentages.

**Figure 5:**
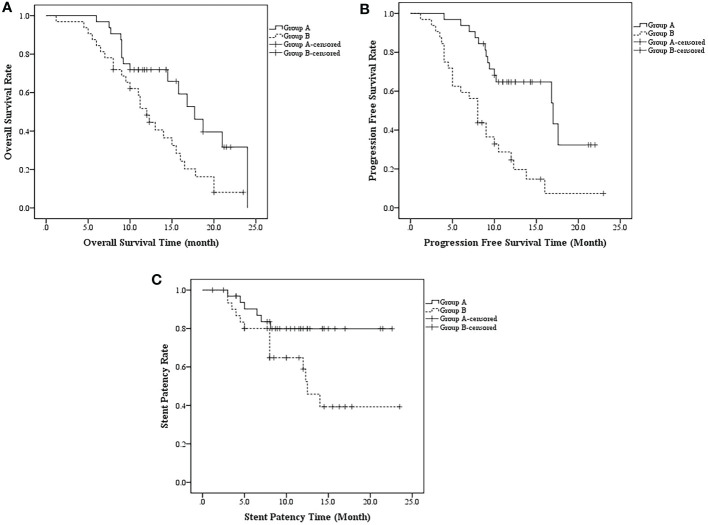
**(A)** The median OS was 17.7 ± 1.7 months (95%CI, 14.3-21.0 months) in group A and 12.0 ± 0.8 months in group B (95%CI, 10.4-13.6 months) (*p* = 0.010); **(B)** the median PFS was 17.0 ± 4.3 (95%CI, 8.5-25.5) and 8.0 ± 0.7 (95%CI, 6.6-9.3) months in groups A and B, respectively (*p* < 0.001); **(C)** The median SPT was not reached in group A and was 12.5 ± 1.1 months in group B (95%CI, 10.3-14.7 months; *p* = 0.028).

In PSM cohorts, median OS, median PFS, median SPT and multivariate analysis were compared between the 2 groups. The median OS was 17.7 ± 1.7 months (95%CI, 14.3-21.0 months) in group A and 12.0 ± 0.8 months in group B (95%CI, 10.4-13.6 months) (*p* = 0.010) (**Figure 5A**). Meanwhile, the median PFS was 17.0 ± 4.3 (95%CI, 8.5-25.5) and 8.0 ± 0.7 (95%CI, 6.6-9.3) months in groups A and B, respectively (*p* < 0.001) (**Figure 5B**). The median SPT was not reached in group A and was 12.5 ± 1.1 months in group B (95%CI, 10.3-14.7 months; *p* = 0.028). (**Figure 5C**)

In univariate analysis, treatment regimen and sex statistically significant at *p* < 0.05 and they were entered a multivariable Cox proportional hazards model. Multivariate analysis found that the treatment regimen and sex were two independent prognostic factors of OS. (**Table 4**)

**Table 4:** Log-rank test and Cox regression analysis of factors potentially related to OS in PSM cohorts.

	32 PSM pairs (n=64)				
		Log-rank test		Multivariate	
Factors	No. of Patients	Median OS (95%CI)	*p-*value	HR (95%CI)	*p-*value
Sex			0.038		0.035
Male Female	59 5	15.8±1.3 (13.3-18.3) 9.5±0.3 (8.9-10.1)		0.350 (0.132-0.930) 1	
Age			0.120		
≥55y	37	12.0±1.8 (8.4-15.6)			
<55y	27	16.8±2.3 (12.3-21.3)			
Treatment regimen			0.010		0.011
Group A	32	17.7±1.7 (14.4-21.0)		0.434 (0.228-0.823)	
Group B	32	12.0±0.8 (10.4-13.6)		1	
Tumor size *(mm)			0.705		
≥10cm	32	13.0±2.3 (8.6-17.4)			
<10cm	32	15.8±0.7 (14.5-17.1)			
Child-Pugh class			0.330		
A	60	15.0±1.5 (12.1-17.9)			
B	4	10.0±3.7 (2.8-17.2)			
Serum AFP level			0.971		
≥400	45	14.5±2.0 (10.7-18.3)			
<400	19	12.3±4.2 (4.2-20.4)			
Extrahepatic metastasis			0.350		
Yes	5	10.0±2.5 (5.1-14.9)			
No	59	15.0±1.5 (11.9-18.0)			

AFP, α-fetoprotein; CI, confidence interval; HR, hazard ratio.

*Tumor size, the maximum diameter of the largest target index lesion.

In group A, 10 patients received pembrolizumab injection (median OS, 16.8 ± 3.7 months; median PFS, 16.8 ± 4.5 months), 9 received toripalimab injection (median OS, 20.0 ± 3.5 months; median PFS, 14.1 ± 3.1 months), 12 received sintilimab injection (median OS, 17.7 ± 2.3 months; median PFS, 17.0 ± 0.0 months), 9 received camrelizumab injection (median OS, 18.0 ± 5.1 months; median PFS, 18.0 ± 6.1 months), and 7 received nivolumab injection (median OS, 19.2 ± 0.4 months; median PFS, 17.6 ± 7.1 months).

### Safety

No serious complications related to interventional treatment, including acute hepatic failure, liver abscess, intraperitoneal bleeding, and radiation hepatitis was observed. The incidence rates of fever, vomiting and upper-abdominal pain were 23.4%, 29.8% and 53.2% in group A, and 27.5%, 22.5% and 60.0% in group B, respectively. They were all resolved after symptomatic treatment.

In 2 groups, all recorded AEs related to systemic treatment are shown in (**Table 5**). Eight (17.0%) and 12 (25.5%) patients occurred 11 and 15 AEs related lenvatinib in group A and B, respectively (*p* = 0.152). Grade 3 diarrhea and hypertension occurred in 1 patient each and led to lenvatinib dose reduction. In group A, 5 (10.6%) patients occurred 5 anti-PD-1 antibody related AEs. Grade 3 immunological enteritis and immunological myocarditis occurred in 1 patient each, and anti-PD-1 antibody injection was ceased permanently.

**Table 5:** Adverse events related to systemic therapy in 2 groups.

	Group A (n=47)	Group B (n=40)	*p*-value
Lenvatinib related AEs			
Diarrhea			
Grade 1-2	3 (6.4)	4 (10.0)	0.698
Grade 3-4	1 (2.1)	0 (0.0)	1.000
Hand-foot skin reaction			
Grade 1-2	2 (4.3)	3 (7.5)	0.658
Grade 3-4	0 (0.0)	0 (0.0)	
Hypertension			
Grade 1-2	4 (8.5)	5 (12.5)	0.727
Grade 3-4	0 (0.0)	1 (2.5)	0.460
Duodenal ulcer			
Grade 1-2	1 (2.1)	0 (0.0)	1.000
Grade 3-4	0 (0.0)	0 (0.0)	
Leukopenia and thrombocytopenia			
Grade 1-2	0 (0.0)	2 (5.0)	0.209
Grade 3-4	0 (0.0)	0 (0.0)	
Anti-PD-1 antibody related AEs			
Immunological hypothyroidism			
Grade 1-2	1 (2.1)		
Grade 3-4	0 (0.0)		
Immunological enteritis			
Grade 1-2	0 (0.0)		
Grade 3-4	1 (2.1)		
Immunological myocarditis			
Grade 1-2	0 (0.0)		
Grade 3-4	1 (2.1)		
Immunological pneumonia			
Grade 1-2	2 (2.4)		
Grade 3-4	0 (4.3)		

Values in parentheses are percentages.

These patients were all relieved by symptomatic treatment (grade 1AEs) and lenvatinib dose reduction and/or anti-PD-1 antibody cease (grade ≥2 AEs). No grade 4 AE occurred, and no patient died of AEs in this study.

## Discussion

This study demonstrated that interventional therapy (^125^I seed strand and PVS implantation plus TACE) combined with systemic therapy (lenvatinib plus anti-PD-1 antibody) is a safe and effective treatment strategy for HCC patients with Vp4 PVTT.

The prognosis of advanced HCC remains poor, especially for patients with PVTT. Furthermore, OS is shorter in patients with Vp4 PVTT than in those with Vp0-3 PVTT (21, 22). The main reason for the poor prognosis is MPV occlusion, which is associated with increased risk of tumor spread, elevated portal venous pressure causing variceal hemorrhage, and decreased portal flow resulting in ascites, jaundice, hepatic encephalopathy, and liver failure (9). However, without treatment the interval between the formation of segmental PVTT and complete obstruction is <6 weeks (23). These previous studies implied that there are two key points in the treatment strategy for patients with Vp4 PVTT: first, restoring the flow of obstructed portal vein; second, inhibiting intrahepatic tumor and PVTT progression.

Luo et al. proposed PVS and ^125^I seed strand which implanted from contralateral branch to MPV combined with TACE treatment for HCC patients with Vp4 PVTT (9). Even though, this interventional treatment strategy prolonged the OS to 9.3 months. The PFS was only 1.8 months and stent occlusion by tumor invasion occurred in 68.1% patients. Based on this interventional technique, a new improvement was made in this study: except for the PVS and ^125^I seed strand which implanted from contralateral branch to MPV, another ^125^I seed strand was implanted into the ipsilateral branch which inhibited the progression of tumor thrombus in ipsilateral branch and prolonged the stent patency time.

According to BCLC stage, sorafenib and lenvatinib were recommended as first-line systemic therapy for patients with HCC and PVTT (7). Zhang et al. conducted a retrospective study that combined sorafenib with interventional therapy proposed by Luo et al. for treating patients with HCC and Vp4 PVTT (10). The median OS and median time to progression (TTP) were 12.3 and 9.0 months, respectively. In recent years, the approval of lenvatinib has provided a new option for patients in BCLC C stage. According to the REFLECT trial, the ORR of lenvatinib is significantly higher than that of sorafenib (6).

More recently, ICI therapy plus anti-VEGF therapy have been recommended as a new effective systemic treatment strategy for patients with advanced HCC. One of the underlying mechanisms is that anti-VEGF therapies can reduce VEGF therapy-mediated immunosuppression within the tumor and its microenvironment may enhance anti-PD-1/PD-L1 efficacy by reversing VEGF-mediated immunosuppression and promoting tumor T-cell infiltration (24). In the IMbrave150 study, ORRs were 33.2% and 13.3% in the atezolizumab-bevacizumab and sorafenib groups, respectively, and OS was significantly longer with atezolizumab-bevacizumab (25). Huang et al. performed a real-world study that analyzed HCC patients with macrovascular tumor thrombus (MVTT) administered lenvatinib plus anti-PD-1 antibodies as first-line treatment (26). This combination therapy resulted in better tumor responses in MVTT (ORR for MVTT, 54.5%) than in intrahepatic tumor (32.8%) and lung metastases (37.5%). Based on these results, whether combined interventional therapy with ICI therapy and TKIs could provide more effective tumor control rate and prolong the OS for patients with unresectable HCC and PVTT?

Cao et al. reported TACE combined with lenvatinib and sintilimab for unresectable HCC with a mOS of 23.6 months and ORR of 46.7% (27). Ju et al. reported TACE combined with apatinib and camrelizumab for advanced HCC with a mOS of 24.8 months which longer than apatinib plus camrelizumab (13.1 months) (28). According to these results, TACE combined TKIs and anti-PD-1 antibody might be an effect combined therapy for advanced HCC and PVTT. However, Vp4 PVTT patients were excluded by these studies. Because MPV occlusion is an important factor which affect safety and prognosis for patients who received TACE or TACE plus systemic therapy (23, 29). In our study, the occluded MPV was restored and kept patent by PVS and ^125^I seed strand. To some extent, based on this interventional treatment regimen, the Vp4 PVTT was down-staged to Vp3. The restoration of MPV provided grantee for normal liver function. Therefore, in our study, TACE combined with lenvatinib and anti-PD-1 antibody could be provided to control tumor progression safely. As a result, patients in group A had significantly better intrahepatic tumor control (55.3% vs 17.5%, *p* < 0.001). And group A patients had significantly longer OS and PFS than group B cases (OS, 17.7 ± 1.7 vs 12.0 ± 0.8 months, *p* = 0.010; PFS, 17.0 ± 4.3 vs 8.0 ± 0.7 months, *p* < 0.001). In group A, 2 patients received liver transplantation and 1 patient received surgical resection. This result implied us that this combined therapy could provide opportunities of surgical treatment for patients with unresectable HCC and Vp4 PVTT.

Furthermore, radiation therapy (RT) has been demonstrated to enhance the priming and effector phases of antitumor-T-cell response, rendering it an attractive therapeutic tool that can be combined with PD-1/PD-L1 inhibitors (30). Two preclinical studies supported the rational combination of RT and PD-1/PD-L1 inhibitors in HCC (31, 32). ^125^I seed strand implantation is a type of endovascular brachytherapy. X-rays and gamma-rays emitted by ^125^I seeds could continuously irradiate the PVTT. In the current study, patients in group A who received anti-PD-1 antibody injection had a significantly lower rate of stent occlusion (12.8% vs 35.0%, *p* = 0.014) and significantly longer median stent patency time (not-reached vs 12.5 ± 1.1 months, *p* = 0.028). Therefore, ^125^I seed may also enhance the therapeutical effect of anti-PD-1 antibodies. More experimental investigations should be conducted to confirm this conclusion.

In addition, 8 (17.0%) and 12 (25.5%) patients occurred 11 and 15 AEs related lenvatinib in group A and B, respectively (*p* = 0.152). The occurrence rate of AEs related to lenvatinib did not increase in patients combined lenvatinib and anti-PD-1 antibodies. No grade 4 AE occurred, and no patient died of AE in this study. Hence, this combined treatment regimen in group A is safe for patients with HCC and Vp4 PVTT.

There were several limitations in the current study. First, this study had a retrospective design, which may affect its generalizability. Second, five different kinds of anti-PD-1 antibody were used in group A, and the sample size was limited, which may affect the survival results. Third, more techniques could be used to evaluate the volume and activity of PVTT more precisely in a future study. Therefore, our next step is to conduct a single-center prospective, randomized, controlled trial to evaluate the long-term efficacy of this encouraging combination therapy in improving survival in HCC patients with Vp4 PVTT.

In conclusion, the interventional therapy (^125^I seed strand and PVS implantation plus TACE) combined with systemic therapy (lenvatinib plus anti-PD-1 antibody) in patients with HCC and Vp4 PVTT is safe and effective. To our knowledge, this is the first report of patients with HCC and Vp4 PVTT administered this combination therapy as first-line treatment.

## Data availability statement

The raw data supporting the conclusions of this article will be made available by the authors, without undue reservation.

## Ethics statement

The studies involving human participants were reviewed and approved by Institutional Review Boards of the Zhongshan Hospital, Fudan University. Written informed consent for participation was not required for this study in accordance with the national legislation and the institutional requirements. Written informed consent was obtained from the individual(s) for the publication of any potentially identifiable images or data included in this article.

## Author contributions

X-DQ and Z-PY conceived and designed the project. Q-XL, L-XL, and J-JL provided administrative support. WZ, J-QM, and M-JY collected the data. Z-HZ, S-NH, and J-ZY analyzed the data and wrote the manuscript. All authors contributed to the article and approved the submitted version.
